# Understanding the Promise and Challenges of Tumor-Agnostic Therapy: Could One Size Really Fit All?

**DOI:** 10.3390/cancers18101568

**Published:** 2026-05-12

**Authors:** Yin M. Myat, Kyaw Z. Thein, Myat M. Han, Manmeet Ahluwalia, Sarbajit Mukherjee, Kyaw L. Aung

**Affiliations:** 1 One Brooklyn Health—Interfaith Medical Center Campus, 1545, Atlantic Avenue, Brooklyn, NY 11213, USA; yinmon.myat@obhny.org; 2 Comprehensive Cancer Centers of Nevada—Central Valley, 3730 S. Eastern Ave., Las Vegas, NV 89169, USA; 3 Kirk Kerkorian School of Medicine, University of Nevada, Las Vegas (UNLV), 4505 S. Maryland Pkwy, Las Vegas, NV 89154, USA; 4 Department of Medicine, Touro University Nevada, 874 American Pacific Dr., Henderson, NV 89014, USA; 5 Avera McKennan Hospital & University Health Center, 1325 S. Cliff Ave., Sioux Falls, SD 57105, USA; 6 Department of Neuro-Oncology, Miami Cancer Institute, Baptist Health South Florida, 8900 N. Kendall Dr., Miami, FL 33176, USA; 7 Department of Gastrointestinal Medical Oncology, Miami Cancer Institute, Baptist Health South Florida, 8900 N. Kendall Dr., Miami, FL 33176, USA; sarbajit.mukherjee@baptisthealth.net

**Keywords:** intratumor heterogeneity, genetic and epigenetic alterations, oncogene addiction theory, tumor-agnostic precision oncology, secondary resistance mechanisms

## Abstract

Tumor-agnostic therapies represent an evolving approach in oncology, shifting from conventional histology-based treatment models to strategies guided by molecular alterations. Regulatory approvals of therapies targeting tumors harboring genomic alterations such as NTRK and RET fusions, BRAF V600E mutation, and those with deficient mismatch repair (dMMR) and a high tumor mutational burden (TMB-H) have demonstrated clinical activity across multiple cancer types. However, responses to these therapies are not uniform across all tumors. This review examines the variability of clinical outcomes across different cancer histologies and the challenges associated with this tumor-agnostic treatment paradigm. Despite sharing the same molecular alterations, some malignancies, including pancreatic and colorectal cancers, demonstrate lower response rates due to tissue-specific resistance mechanisms such as bypass signaling pathways and co-occurring genomic alterations. We discuss how these biological differences influence treatment response and their implications for future drug development and clinical trial design. Addressing these biological and clinical complexities will be essential to optimize the use of tumor-agnostic therapies across diverse cancer types.

## Simple Summary

Tumor-agnostic therapies, driven by molecular targets rather than tissue of origin, mark an important shift towards precision oncology. The accelerating approvals of tumor-agnostic treatments encompassing multiple cancer types in recent years by the US Food and Drug Administration show that this treatment paradigm is benefiting cancer patients in clinics. Yet, variable responses observed with these treatments and heterogeneity in secondary resistance mechanisms highlight the challenges posed by the complexity of the underlying tumor biology. This review explores the clinical promise of tumor-agnostic therapies, underscores their limitations, and discusses the necessity of integrating tissue-specific factors into future development.

## 1. Introduction

A deep understanding of oncogenic drivers has enabled the development of tumor-agnostic therapies and enhanced our ability to provide personalized care. This success raises hope and fuels efforts to obtain more tumor-agnostic therapy approvals in an attempt to move away from conventional histology-driven management approaches. As the field moves forward, it is of critical importance to understand the real benefits brought to cancer patients by this new tissue-agnostic treatment paradigm and the challenges we face for its wider applications.

The current premise of tumor-agnostic therapy is based upon the commonality of oncogenic driver mutations and tumor immune escape mechanisms shared between different anatomical and histological tumor types. Despite numerous genetic and epigenetic alterations acquired during tumorigenesis and progression, tumor cells are most dependent on a select few alterations in oncogenes rather than the summation of all individual genomic alterations [1]. And, notably, these few oncogenic alterations are shared by multiple tumor types. This is best exemplified by oncogenic BRAF mutations [2]. The success of BRAF inhibitors in the development of tumor-agnostic therapies validated the oncogene addiction theory and signaled hope for future tissue-agnostic drug development. Subsequently, therapies targeting NTRK and RET fusions demonstrated efficacy, showcasing the potential of a tumor-agnostic approach for rare alterations and histologies [3,4,5,6]. Furthermore, we now know that there is a commonality in tumor immune escape mechanisms best exemplified by PD-1/PD-L1 pathway activation seen across different cancers [7]. Therapeutic exploitation of this biological insight has led to tumor-agnostic approvals of immune checkpoint inhibitors (ICIs) in patients with microsatellite instability-high (MSI-H) solid tumors and tumor mutation burden-high (TMB-H) solid tumors. The mutational expression of certain alterations may, however, exist as a continuous spectrum, with unique biological mechanisms that contribute to heterogeneous efficacy. The novel lessons of antibody drug conjugates (ADCs) targeting HER2-positive solid tumors present another avenue to deepen our understanding of tumor-agnostic precision oncology.

Although these developments are undoubtedly encouraging, to realize the true potential of tumor-agnostic therapy, it would be a mistake to ignore tissue-specific factors that have intricate interplays with tumor-agnostic common biological mechanisms. Primary and secondary resistance to tissue-agnostic therapies remains a major challenge in achieving long-term tumor control, and past lessons have shown that resistance mechanisms to a particular agnostic therapy could be different among different tumor types [8,9]. Thus, understanding the differences in responses and mechanisms of resistance within the tissue-specific context is essential for the development of more effective histology-agnostic therapies.

**Figure 1:**
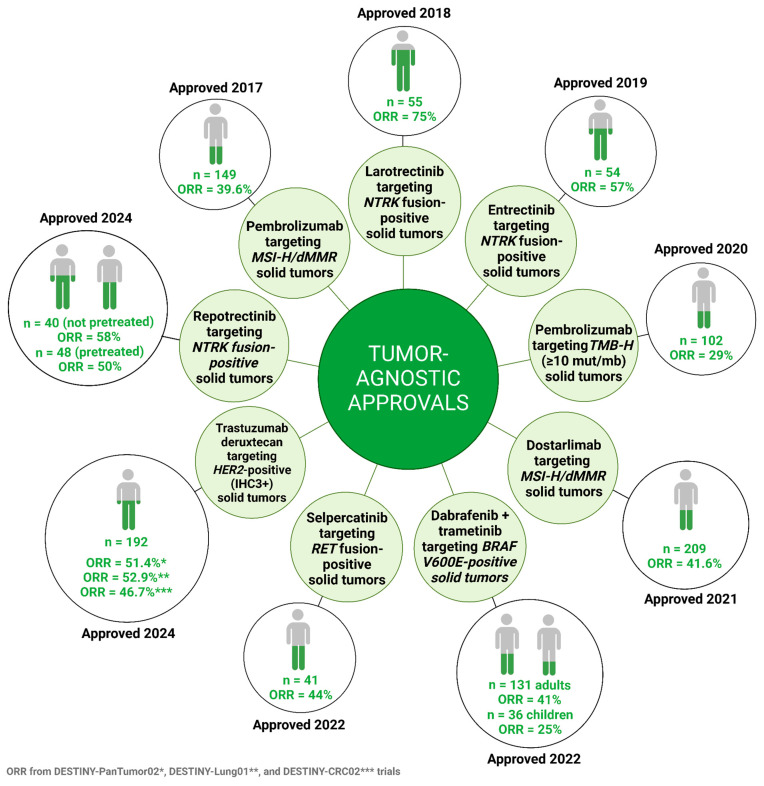
Overview of FDA-approved tumor-agnostic therapies. Created in BioRender. Thein, K. (2026) https://BioRender.com/an440nb.

There are now several tumor-agnostic treatments indicated for patients with solid tumors (summarized in Figure 1 and Table 1 below). The acceleration seen in getting these drugs approved by the US Food and Drug Administration (FDA) for patients with unique molecular aberrations is encouraging and has undoubtedly benefited cancer patients. As this tumor-agnostic treatment paradigm gains momentum, taking a pause and dissecting the key lessons we learned from developing these drugs is necessary to improve future drug development. Herein we focus our discussion on two main lessons: heterogeneity in efficacy reflecting the differences in primary resistance mechanisms and heterogeneity in secondary resistance mechanisms. While our understanding of heterogeneity in primary resistance mechanisms remains quite limited, the landscape of secondary resistance mechanisms to tumor-agnostic therapies keeps evolving. Better understanding of primary and secondary resistance mechanisms will optimize the benefits derived from tumor-agnostic treatments.

This narrative review was conducted to evaluate the current landscape of tumor-agnostic therapies, with a focus on clinical efficacy and mechanisms of primary and secondary resistance across different tumor types. A structured literature search was performed using PubMed and Google Scholar to identify relevant studies published up to April 2026. Additional data were obtained from pivotal clinical trial reports, United States Food and Drug Administration regulatory documents, and conference abstracts when peer-reviewed publications were not yet available.

Search terms included combinations of: “tumor-agnostic therapy”, “microsatellite instability-high”, “tumor mutational burden-high”, “rearranged during transfection gene fusion”, “pembrolizumab”, “dostarlimab”, “dabrafenib”, “trametinib”, “selpercatinib”, “repotrectinib”, “trastuzumab deruxtecan”, and “immune checkpoint inhibitor”. Reference lists of key review articles and landmark clinical trials were also screened using backward citation tracking to identify additional relevant studies.

Studies were included if they reported clinical outcomes, including objective response rate, duration of response, progression-free survival, or overall survival, or if they provided mechanistic insights into resistance to tumor-agnostic therapies across solid tumors. Both prospective clinical trials and relevant translational or retrospective studies were included to provide biological and clinical context.

Given the heterogeneity in study design, patient populations, biomarker definitions, and outcome measures across tumor types, findings were synthesized qualitatively. Particular emphasis was placed on comparing therapeutic efficacy and resistance patterns across histologies sharing the same molecular alteration in order to highlight tissue-specific variability within the tumor-agnostic treatment framework.

## 2. Heterogeneity in Efficacy

### 2.1. BRAF Inhibitors

**Figure 2:**
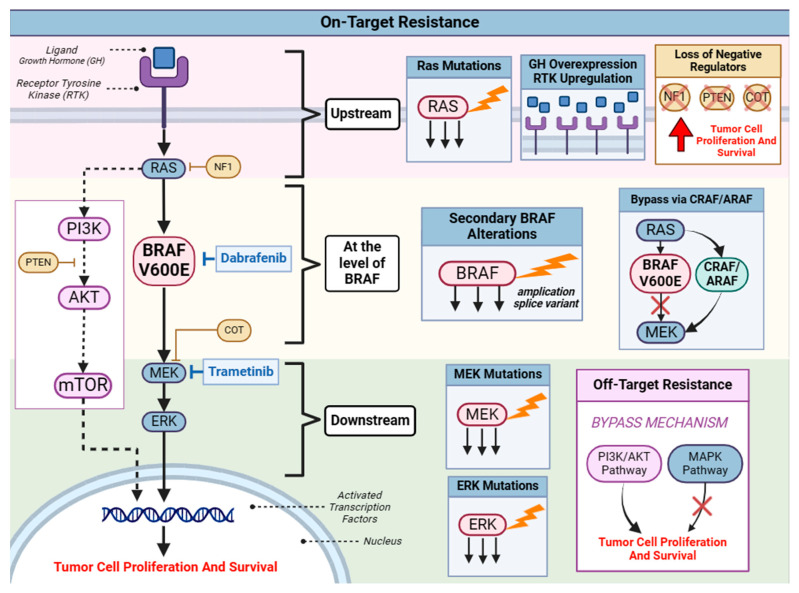
Resistance concerning the MAPK signaling pathway: This figure illustrates the common resistance mechanisms concerning dabrafenib and trametinib targeting BRAF V600E and MEK, respectively. The on-target resistance mechanisms primarily involve hyperproliferation of the MAPK signaling pathway, although other pathways or factors may also contribute. (a) On-target resistance upstream to BRAF: RAS mutations, including KRAS co-mutations, as well as growth hormone overexpression and upregulation of receptor tyrosine kinase receptors, may result in resistance. (b) On-target resistance at the level of BRAF: BRAF molecules may acquire additional alterations, such as amplification or splice variants. Despite BRAF inhibition, the MAPK pathway may still be activated due to bypass mechanisms involving CRAF and ARAF. (c) On-target resistance downstream to BRAF: Mutated MEK may contribute to resistance in patients receiving dabrafenib-plus-trametinib regimens. In particular, ERK is downstream to both BRAF and MEK, thus leading to the paradoxical activation of the MAPK pathway. This activation can lead to reduced efficacy of BRAF and MEK inhibitors due to continued signaling through the MAPK pathway, despite treatment with these targeted therapies. Created in BioRender. Thein, K. (2026) https://BioRender.com/an440nb.

*BRAF* somatic mutations were present in 3–7% of human cancers [19]. The vast majority (>90%) of *BRAF* mutations are *BRAF^V600E^* mutations located on exon 11 or 15. *BRAF^V600E^* has been found in approximately 50% of melanomas, 40% of papillary thyroid cancers, 10% of colorectal cancers (CRCs), and 5% of lung cancers [20]. Vemurafenib became the first BRAF inhibitor approved by the FDA for *BRAF^V600E^*-positive metastatic melanomas. The use of BRAF inhibitors has since expanded [21]. Dabrafenib plus trametinib (D + T) represents the first *BRAF* inhibitor combination approved for tissue-agnostic purposes. The FDA approved this combination for *BRAF^V600E^*-positive unresectable or metastatic cancers based on the data from the NCI-MATCH, ROAR, and CTMT212X2101 clinical trials. Although treatment response was seen across different tumor types, there was a wide variation in objective response rates and median durations of response (MDRs) between cancer types, with MDRs ranging from 1.8 months to 30 months, as shown in Figure 2 below (data summarized in the Supplementary Table S1). Except for one patient with mandibular ameloblastoma and five patients with low-grade serous ovarian carcinoma, MDRs observed in other cancer types were rather modest. It is noteworthy that biliary tract cancers had the lowest MDR at 1.8 months. For this D + T tumor-agnostic approval, CRC was notably excluded due to its known intrinsic resistance [15], highlighting the importance of understanding the primary resistance mechanisms within tissue-specific contexts. Currently, encorafenib-plus-cetuximab combination therapy is approved for *BRAF^V600E^*-positive mCRC in the second/third-line setting based on the BEACON trial [22]. Similarly, the BREAKWATER trial observed clinical benefits of encorafenib, cetuximab, and mFOLFOX6 chemotherapy regimens in the same cohort, including the first-line setting [23].

### 2.2. Inhibitors of NTRK and RET Gene Fusions

The *NTRK* inhibitors larotrectinib, entrectinib and repotrectinib, as well as a RET inhibitor, selpercatinib, are approved for tumor-agnostic use [11,12,16,18]. Larotrectinib was approved for refractory NTRK fusion-positive solid tumors in adults and children based on three clinical trials: LOXO-TRK-14001 (phase I), NAVIGATE (phase I/II), and SCOUT (phase II). Entrectinib, another NTRK inhibitor that has activity against other RTKs, such as ROS1 and ALK, was approved for refractory NTRK fusion-positive solid tumors. Thirdly, repotrectinib was granted accelerated approval for NTRK gene fusion-positive solid tumors based on data from the TRIDENT-1 phase I/II trial [24]. Selpercatinib was approved for advanced or metastatic RET fusion-positive solid tumors based on LIBRETTO-001, a global phase I/II trial featuring a pan-cancer patient population [25]. Although high ORRs were observed across multiple tumor types, MDRs again varied. Relatively low MDRs were observed with larotrectinib in CRC and pancreatic cancer (Figure S1A) [26,27]. The MDRs achieved with entrectinib in these cancers [28], however, were higher, highlighting potential differences in patient selection and/or heterogeneity, as well as differences in mechanisms of action of these two drugs (Figure S1B). However, as patient numbers included in these studies are small, it is challenging to draw any definitive conclusions. Overall, despite the variability, quite impressive MDRs are observed with entrectinib across multiple tumor types in NTRK fusion-positive tumors (Figure S1B). This was also the case with selpercatinib in RET fusion-positive tumors (Figure S1C) [29,30,31]. These data collectively indicate that NTRK and RET inhibitors truly represent the tumor-agnostic paradigm. The efficacy of these fusion inhibitors in different cancer types is summarized in Table S1.

### 2.3. Immune Checkpoint Inhibitors

#### dMMR/MSI-H Solid Tumors

Pembrolizumab was the first immune checkpoint inhibitor that was FDA-approved for tumor-agnostic use for patients with advanced dMMR/MSI-H solid tumors in the refractory setting. The initial approval was based on the results from five phase II clinical trials: KEYNOTE-016, KEYNOTE-164, KEYNOTE-012, KEYNOTE-028, and KEYNOTE-158. Subsequently, it received the full approval for the same indication based on the results from three phase II trials (KEYNOTE-164, KEYNOTE-158, AND KEYNOTE-051) [10,32,33]. A total of 504 patients with >30 different cancer types were treated with pembrolizumab in these studies. The overall ORR was 33.3%; 10.3% were CR and 23% were PR [34]. Although ORRs were quite similar across tumor types, with the exception of pancreatic cancer, there clearly were some variations. Patients with endometrial cancer had an ORR of 48.5%, whereas those with pancreatic cancer had the lowest ORR of 18.2% (Figure S2). Additionally, there was variation in the mPFS data. Small intestinal cancer had the longest mPFS of 23.4 months, while those with pancreatic cancer had the lowest mFS of 4.2 months [32]. Dostarlimab, another PD-1 inhibitor, also recently received histology-agnostic approval for patients with dMMR solid tumors. With this drug, ORR differed between CRC patients and non-CRC patients: 36.2% vs. 43.2% (Figure S3) [35,36]. Again, four patients with dMMR pancreatic cancer in the dostarlimab study failed to achieve an objective response.

### 2.4. TMB-H Solid Tumors

The approval of tumor-agonistic pembrolizumab for patients with advanced TMB-H (≥10 mut/Mb) solid tumors came after the results of a pan-cancer cohort of 102 patients treated with pembrolizumab monotherapy across eight different cancer types in the phase II KEYNOTE-158 trial [37]. An ORR of 29.45%, including 4 CR and 26 PR, was observed in the TMB-H population. Approximately a third of the cohort (34 out of 102 patients) were patients with small cell lung cancer (SCLC). ORRs differed widely across treated tumor types (Figure S4) [37,38]. However, patients with TMB-H status had higher response rates compared to non-TMB-H cancers, in general. This was not the case for those with anal cancer and mesothelioma, although these results should be interpreted with caution due to the low number of patients. In the real-world setting, patients with TMB-H (≥20 mut/Mb) cancers treated with multiple types of ICIs had a higher pan-cancer ORR of 64.1%. This pan-cancer cohort consisted of 56 patients with melanoma, 24 patients with EC, 20 patients with lung adenocarcinoma, 8 patients with CRC, and patients with multiple other tumor types of less frequency [39]. Compared to KEYNOTE-158, this study had a completely different composition of cancer types, some of which were not included in the trial. In addition, the TMB-H cutoff used was different. Still, heterogeneity in ORRs across different cancer types was observed (Figure S4).

### 2.5. Antibody Drug Conjugate (ADC)

In 2024, trastuzumab deruxtecan (T-Dxd) was approved for human epidermal growth factor receptor 2 (HER2)-positive advanced or metastatic solid tumors. HER2-positive status was based on an immunohistochemistry (IHC) 3+ score [40]. Most patients had a response regardless of cancer histology (Supplementary Table S1) [41,42,43]. The DESTINY-PanTumor02 trial further elucidates the variable response rates observed across different cancer types. Patients with breast cancer and other solid tumors of the reproductive tract (endometrium, cervix, ovaries) exhibit superior efficacy, likely attributable to increased HER2 expression [44]. However, certain cancer types, such as pancreatic cancer, show poor efficacy. MDRs differed among these tumor types, as noted with other tumor-agnostic treatments (Figure S5).

## 3. Heterogeneity in Resistance Mechanisms

### 3.1. BRAF Inhibitors

**Figure 3:**
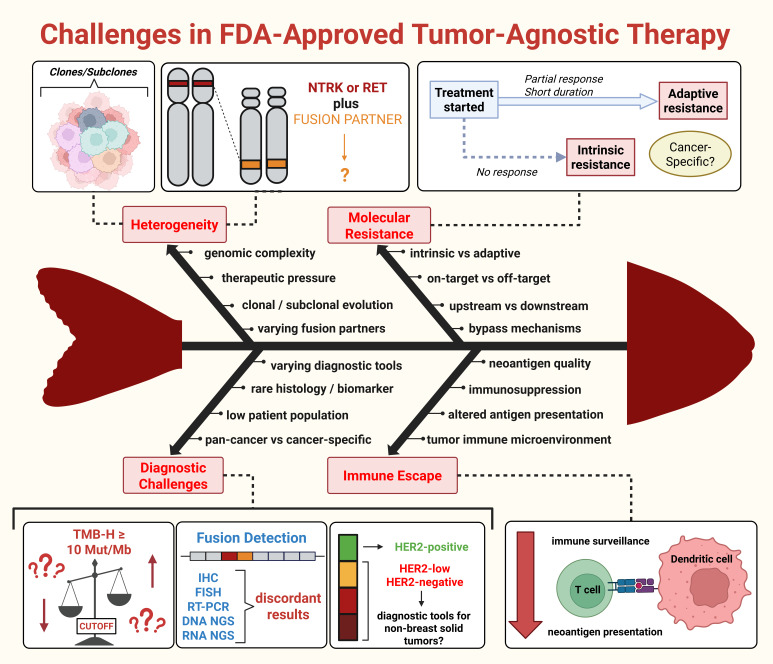
Challenges of approved tumor-agnostic therapies. Description: This figure provides an overview of the lessons and challenges surrounding the approved tissue-agnostic treatments that are discussed in this review. (a) Heterogeneity: Variations in efficacy among different cancer types can be attributed to genetic diversity within clonal and subclonal populations. Mutational evolution may occur post-treatment due to therapeutic pressure. Fusion-gene-positive solid tumors may exhibit heterogeneous results due to differences in fusion partners. (b) Molecular resistance: Patients may exhibit short-lived or partial responses, often attributed to adaptive resistance. Intrinsic resistance may cause some patients to be non-responders. Investigating cancer-specific mechanisms contributing to resistance is an evolving research area. (c) Diagnostic challenges: Establishing an optimal cutoff for tumor mutational burden-high (TMB-H) remains controversial, although efforts are underway to standardize ≥10 mut/Mb. Upward and downward arrows indicate the heterogeneous cutoff thresholds defining TMB-H. Fusion-gene-positive solid tumors have various diagnostic tools, but lack of standardization may lead to discordant results. Detection of HER2-positive cancers is challenging due to varying HER2 expression, and there are no established guidelines for diagnosing non-breast cancers. (d) Immune escape: Effective immunotherapy relies on the interaction between immune and tumor cells. Immune escape occurs when the immune system fails to prevent oncogenesis or emerging resistance mechanisms. This results in decreased neoantigen presentation and ultimately reduces immune surveillance, as represented by the downward arrow. Abbreviations: Colorectal cancer (CRC), deficient mismatch repair (dMMR), fluorescence in situ hybridization (FISH), human epidermal growth factor receptor 2 (HER2), immunohistochemistry (IHC), microsatellite instability-high (MSI-H), mutations per megabase (mut/Mb), neurotrophic tyrosine receptor kinase (NTRK), next-generation sequencing (NGS), objective response rate (ORR), patient population (n), REarranged during Transfection (RET), Reverse Transcription Polymerase Chain Reaction (RT-PCR), tumor mutational burden-high (TMB-H). Created in BioRender. Thein, K. (2026) https://BioRender.com/an440nb.

Despite the fact that dabrafenib plus trametinib has demonstrated favorable efficacy in melanoma, thyroid cancer, and NSCLC, resistance still occurs upstream, downstream, and at the level of inhibition [Figure 3]. While dysregulation of the MAPK signaling pathway represents the most common mechanism of resistance to *BRAF^V600E^* inhibition, other tumor-type-specific resistance mechanisms were also observed.

*EGFR* upregulation represents a unique resistance mechanism for CRC patients receiving a BRAF inhibitor. This is due to a higher concentration of *EGFR* in CRC due to its epithelial origin. In comparison, melanoma cells are derived from neural crest cells, which have lower *EGFR* expression [20]. *EGFR* hyperactivation can lead to a feedback loop of the MAPK pathway, leading to tumor proliferation and growth. Corcoran and colleagues performed an additional pharmacodynamic analysis on 17 out of the 43 patients with *BRAF^V600E^*-positive mCRC (ORR = 12%) who received D + T combination treatment. Nine evaluable biopsies demonstrated a median 37% decrease in phosphorylated ERK (pERK) levels 15 days after starting treatment compared to pretreatment levels. In comparison, patients with melanoma who received dabrafenib monotherapy had a median 75% decrease in pERK [45]. The differences seen between these two cancer types suggest that the resistance in mCRC is primarily insufficient blockage of the MAPK pathway. This allows the negative feedback reactivation of *RAS* and *CRAF* due to intact downstream *ERK*. Although this usually represents physiological feedback, *BRAF*-mutated cells are able to bypass regulatory mechanisms [46]. Furthermore, hyperactivation of the *PI3K/AKT* pathway may have a greater role in mCRC resistance compared to melanoma. In cell lines harboring loss of *PTEN* alterations or *PIK3CA* mutations, a greater level of resistance was observed [47]. Both *PTEN* and *PIK3CA* alterations can lead to activation of *AKT*, thereby bypassing the usual MAPK pathway. Patients with *PTEN* alterations did not have a statistically different objective response rate compared to those without *PTEN* alterations. However, those with *PTEN* mutations had a shorter median progression-free survival (PFS) (18.3 weeks) compared to those without *PTEN* alterations (32.1 weeks) [48].

In NCLC, 22% of patients with *BRAF^V600E^* mutation had additional molecular alterations. Anecdotally, four patients with alterations in the PI3K/AKT pathway had a shorter mOS of 5.4 months, suggesting alterations in the PI3K/AKT pathway as a primary resistance mechanism to BRAF inhibition. Other resistance mechanisms identified include alterations in upstream components such as *KRAS* co-mutations, resulting in downstream *MEK* activation. *KRAS* mutations are the most frequent mutations in lung adenocarcinomas [49]. The resistance mechanisms observed with drugs targeting the MAPK pathway are summarized below in Figure 2.

### 3.2. Inhibiting NTRK and RET Fusion Genes

Both approved NTRK and RET fusion inhibitors have shown promising efficacy; however, most patients do not have a complete response, suggesting that resistance is still an issue. Although primary resistance mechanisms are not well understood, many patients experience acquired or secondary resistance through on-target mechanisms that affect drug binding or off-target mechanisms affecting other pathways. While these mechanisms are not tumor-type-specific, they encompass various unique heterogeneous mechanisms. In TRK fusion-positive solid tumors, on-target mechanisms include alterations to the kinase domain [50].

In RET fusion-positive cancers, on-target resistance is mainly driven by secondary RET kinase domain mutations, particularly solvent-front (e.g., G810) and other non-gatekeeper alterations that reduce drug binding through steric hindrance. Despite being designed to overcome classic gatekeeper mutations, these agents remain vulnerable to such structural changes [51]. On the other hand, off-target resistance mechanisms include activation of other pathways, such as the MAPK signaling pathway. For example, BRAF, MET, and EGFR alterations may play a role. Cocco and colleagues demonstrated that some patients had short-lived responses due to acquired BRAF and MET amplifications [52]. Off-target resistance may also involve the PI3K/AKT and insulin growth factor receptor type 1 (IGF1R) pathways [53]. Some patients may also harbor KRAS, ALK, and ROS1 co-mutations. Wang and colleagues found that different RTK fusions demonstrated different co-mutation rates. For instance, RET fusion-positive cancers had a higher frequency of PTEN co-mutations, whereas ROS1 fusion-positive cancers had a higher frequency of RB1 co-mutations [51].

Currently, the frequency and impact of co-mutations in RET fusion-positive solid tumors have not been fully explored. However, co-mutations involving the MAPK signaling pathway and other tyrosine kinase families (such as EGFR and ALK) are not commonly seen with RET fusions [54,55]. Interestingly, Rich and colleagues found that patients with NSCLC that harbored subclonal CCDC6-RET or NCOA4-RET gene fusions experienced acquired resistance. The authors suggest that this subclonal acquisition of RET fusions is separate from other resistance mechanisms [56].

Resistance to NTRK inhibition commonly arises through on-target kinase domain mutations, particularly solvent-front and xDFG alterations. Solvent-front mutations occur within the hydrophilic, solvent-exposed region of the nucleotide-binding loop, whereas xDFG mutations affect the activation loop of the kinase domain, reducing tyrosine kinase inhibitor (TKI) binding through steric hindrance. Recurrent acquired mutations—including *NTRK1* G595R, G667C, F589L, and G667S, as well as *NTRK3* G696A and G623R—have been identified in tumors resistant to first-generation TRK inhibitors such as entrectinib and larotrectinib [57,58,59,60].

Second- and third-generation TRK inhibitors, such as repotrectinib and selitrectinib, have demonstrated durable activity against kinase domain resistance mutations. In parallel, select multikinase inhibitors retain activity against specific resistant variants, although their overall clinical efficacy remains limited. For example, taletrectinib, a next-generation TKI targeting ROS1 and NTRK1/3, has shown preclinical activity against multiple resistance mutations, including G595L, L564H, F646I, and D679G [61]. Beyond next-generation inhibitors, combination strategies targeting bypass signaling pathways—such as dual TRK and MET inhibition—have shown preliminary efficacy in overcoming off-target resistance [62]. Similarly, therapeutic approaches in RET fusion-positive cancers are evolving from first-generation multikinase inhibitors toward more selective RET inhibitors capable of targeting resistance mutations, including pralsetinib, which demonstrates activity against gatekeeper mutations (RET V804L/M) [63]. Collectively, these findings highlight the need for adaptive combination strategies targeting multiple oncogenic drivers to effectively suppress heterogeneous resistant clones arising from dynamic tumor evolution and acquired fusions.

### 3.3. Immune Checkpoint Inhibitors

Although dMMR and MSI status are greatly interlinked, they are not interchangeable. For instance, among 149 patients, 47 had dMMR status, 60 had MSH-H, and 42 harbored both biomarkers [64]. Brooksbank and Martin suggest that patients with dMMR/MSS tumors may also experience resistance [65]. However, more data is required to determine the difference in response rate between dMMR/MSS and dMMR/MSI-H cancers.

Among 86 patients across 12 different MSI-H cancer types, 14% of patients demonstrated primary resistance based on progressive radiologic features. In comparison, only five patients experienced acquired resistance. These included two patients with brain cancer and one patient with bone-related malignancy. Approximately 20–25% of patients with dMMR/MSI-H mCRC are refractory to ICI treatment [66]. The heterogeneous efficacy of immunotherapy may be explained by the dynamic balance of cancer cells and immune cells in the tumor microenvironment (TME). ICI resistance is often due to interference with CD8+ T cells. The tumor microenvironment may also explain some of the differences in efficacy and survival depending on microsatellite stability status in CRC. This includes alterations involving regulatory T cells (Tregs), myeloid-derived suppressor cells (MDSCs), and tumor-associated macrophages (TAMs) [66]. MSI-H CRCs with APM mutations also had a lower level of CD8+ T cells [67]. Patients with MSI-H CRC also tend to demonstrate an increased rate of acquiring copy number aberrations (CNAs) compared to MSS CRC. These differences may be due to immune escape via genetic alterations in the APM and the WNT/beta-catenin pathway. One analysis found that MSI-H CRCs had a higher number of APM mutations as well as HLA alterations, resulting in selective pressure and subclonal heterogeneity [67]. Interestingly, a study by Rodig and colleagues suggests that loss of at least 50% MHC class I expression did not contribute to pembrolizumab primary resistance in metastatic melanoma patients. However, it did result in resistance to cytotoxic T-lymphocyte–associated antigen 4 (CTLA-4) inhibitors. On the other hand, the presence of MHC class II molecules on melanoma cells was associated with greater response to PD-1 inhibitors compared to CTLA-4 inhibitors [68]. The authors suggest that interferon-γ (IFN-γ) pathways, which are associated with MHC class II, are important for innate immunity when MHC class I is dysfunctional or absent.

Resistance may also occur due to heterogeneity from co-mutations. In The Cancer Genome Atlas Program (TCGA) database, ICI-treated MSI-H CRCs demonstrated a higher expression of twenty frequent mutations compared to MSS tumors. However, *APC* and *TP53* mutations were more common in MSS CRC. No differences in the frequency of *Kirsten rat sarcoma virus* (*KRAS*) mutations between the two cohorts were found. *POLE* and *DNA polymerase delta 1* (*POLD1*) mutations are more common in patients with MSI-H tumors compared to MSS tumors. However, their overall frequency is low. For instance, in the GARNET trial, a total of 347 patients with dMMR or MSI-H or *POLE*-mutated cancers were evaluated. This included only five patients with dMMR solid tumors who harbored a *POLE* co-mutation. Among 11 total patients with the *POLE* mutation, the response rate was 54.5% (95% CI 23.4–83.3) and mPFS was 19.5 months (9% CI 1.2–NR). In comparison, those with dMMR solid tumors without *POLE* co-mutations had an ORR of 44% and mPFS of 6.9 months [69]. Some patients harbor *POLE* mutations in MSS cancers. Given the low frequency of this mutation, it is difficult to determine how *POLE* co-mutations affect response to pembrolizumab. Furthermore, *BRAF^V600E^* and *KRAS* co-mutations may also occur. Although they are more common in MSS CRC, they may still be found in MSI-H tumors at lower frequencies. Patients who have the *BRAF* co-mutation have a poorer prognosis [70]. In EC, MSI-H status is frequently associated with an increased population of frameshift mutations. This results in a heavier neoantigen load that can predict ICI response. In contrast, patients with *POLE*-mutated MSS EC may not experience the same efficacy [71]. This suggests that, in addition to MSI status, other biomarkers should still be taken into consideration.

### 3.4. Trastuzumab Deruxtecan

#### Co-Alterations

HER2 amplification is required for the early stage of tumorigenesis. Interference with this may confer susceptibility to resistance [72]. This is commonly due to co-mutations affecting the MAPK-, PI3K/AKT-, and IGF1R-related pathways. Based on a cohort from TCGA, approximately 31% and 40% of HER2 breast cancers harbored PIK3CA and TP53 mutations, respectively [73]. Colorectal cancers with RAS and RAF co-mutations have been associated with greater intratumor heterogeneity. This may also explain why HER2-expressing CRCs have poor response rates [74]. Interestingly, Singh and colleagues found that CRC patients were resistant to various trastuzumab-based regimens but remained responsive to T-Dxd [75]. Overexpression of the HER2 dimeric partners may also result in resistance. This most commonly involves HER1 and HER3 [66]. However, upregulation of other receptors, such as MET, FGFR, and AXL, also contributes [76,77]. A minority of resistance mechanisms involve structural mutations of the HER2 receptor. This impairs HER2 receptor binding. Cancer type determines which specific HER2 mutations are more dominant. For instance, point mutations in the extracellular domain were more common in cancers of the liver, pancreas, cervix, urinary tract, and skin. In contrast, insertion mutations affecting the kinase domain were more frequent in lung and ovarian cancer [72]. Neupane and colleagues also suggest that, in theory, irinotecan pretreatment may contribute to deruxtecan cross-resistance. However, irinotecan is not commonly used for breast cancer. HER2 expression may also contribute to heterogeneity. For instance, the loss of HER2 expression has been associated with resistance [78].

### 3.5. HER2 Heterogeneity

The level of HER2 expression is a continuous spectrum rather than a dichotomous value. HER2-positive tumor cells may have neighboring cells that have minimal or no HER2 receptors on their surface. Cancers with higher HER2 expression are more aggressive. In contrast, HER2-low status was more frequent in low-grade tumors [79], although these cancers are difficult to target. For instance, cancers with high HER2 expression (HER2-positive) frequently confer a superior response rate compared to cancers with low HER2 expression. Thus, neighboring tumor cells may have varying HER2 status that affects efficacy. Lin and colleagues reported superior overall survival in HER2-low versus HER2-negative BC, but conflicting findings exist [73]. The heterogeneity in HER2-amplified non-breast cancers is poorly documented. While loss of HER2 expression is a resistance mechanism, some tumors may acquire HER2 expression over time [74]. The HER2 heterogeneity association with treatment response differs based on cancer type. Thus, Valenza and colleagues suggest that HER2 heterogeneity is not an indicator of agnostic benefit [80].

HER2 intratumor heterogeneity has been reported in approximately 10% of breast cancers [81]. A subset of patients with HER2-amplified breast cancer had intratumor heterogeneity, which conferred decreased survival and poor prognosis. Heterogeneity is more commonly found in GC compared to BC [82]. However, the data for pan-cancer cohorts is still lacking. It is uncertain whether this is due to loss of HER2 expression or whether unstable HER2-negative subclones acquire amplification.

## 4. Future Perspectives

There is an increasing number of agnostic treatments with more targetable biomarkers: KRAS, fibroblast growth receptor (FGFR) and other targets are prime examples.

One clinically relevant KRAS subtype is KRAS G12C, which has emerged as the most successfully drugged mutant following the discovery of the switch II pocket, an allosteric binding site accessible during the GDP-bound state of KRAS. This vulnerability is linked to the unique GDP–GTP cycling dynamics of KRAS G12C, enabling covalent trapping of the inactive form [83]. Targeted covalent inhibitors such as sotorasib and adagrasib have demonstrated clinical activity, particularly in non-small cell lung cancer (NSCLC), with more variable responses observed in non-lung malignancies. Notably, sotorasib became the first KRAS G12C inhibitor approved for previously treated advanced NSCLC on 28 May 2021, marking a major milestone in targeting KRAS-driven oncogenesis [84]. Adagrasib was later approved for patients with *KRAS^G12C^*-positive NSCLC on 12 December 2022, based on the KRYSTAL-1 phase II trial. Divarasib is a highly potent *KRAS^G12C^* inhibitor that is currently in ongoing phase 1 trials for advanced solid tumors harboring the alteration. It has shown favorable responses for both NSCLC and CRC. In the NSCLC cohort, the confirmed ORR was 53.4%, the mPFS was 14 months, and the mOS was 13.1 months. Patients with CRC also had an impressive response rate of 29.1% [85].

Daraxonrasib (RMC-6236) is an oral, potent, and “molecular glue” RAS (ON) multi-selective inhibitor targeting KRAS G12X (including G12D/V/C/R/S/A), G13X, and Q61X mutations. It has shown activity in patients with metastatic pancreatic adenocarcinoma (mPDAC) [86]. It demonstrated significant efficacy in the Phase 3 RASolute 302 trial for previously treated metastatic pancreatic cancer (PDAC), with a median OS of 13.2 months vs. 6.7 for chemo [87].

FGFR alterations represent another promising tumor-agnostic target within the tyrosine kinase family, identified across multiple malignancies. Erdafitinib, a pan-FGFR inhibitor targeting FGFR1–3 alterations, has demonstrated encouraging activity in the phase II RAGNAR trial, with an objective response rate of 64% across diverse tumor types [88]. Additional FGFR inhibitors, including pemigatinib and infigratinib [89,90], further support the therapeutic potential of FGFR-directed strategies in biomarker-selected populations.

Tumor-agnostic treatments are expanding with new biomarkers and targeting new pathways. Apart from cellular proliferation and survival pathways, there are agents targeting DNA repair mechanisms and the cell death pathway. Targeting homologous recombination deficiency (HRD) with Poly (ADP-ribose) polymerase (PARP) inhibitors represents another tumor-agnostic strategy, particularly in tumors with BRCA1/2 and related defects, though resistance mechanisms such as restoration of homologous recombination continue to emerge. Among PARP inhibitors, olaparib has the most robust and extensive evidence across tumor types, supported by landmark trials such as OlympiAD and PROfound, while agents including niraparib, rucaparib, and talazoparib have further expanded the role of PARP inhibition in HR-deficient malignancies [91,92,93,94,95]. Notably, recent studies have extended the benefit beyond BRCA-mutated tumors to broader HRD-positive populations, although variability in response highlights the need for improved biomarker selection and combination strategies.

In parallel, therapies targeting cell death pathways offer promising strategies to overcome resistance by directly modulating both apoptotic and non-apoptotic mechanisms. Rezatapopt, an oral p53 reactivator targeting TP53 Y220C-mutated tumors, is currently under evaluation in a phase I study. In a cohort of 77 heavily pretreated patients, rezatapopt demonstrated an objective response rate of 20% overall and 30% in those with KRAS wild-type tumors, with activity observed across multiple tumor types, including ovarian and breast cancers [96]. Collectively, these findings highlight a shift toward biomarker-driven and combination-based strategies, integrating genomic and functional profiling to expand the scope of tumor-agnostic precision oncology.

## 5. Conclusions

The promises and dilemmas of tumor-agnostic therapy represent the new paradigm for unraveling the secrets of precision oncology. Understanding the biological nature of genomic alterations and their role in intratumor heterogeneity and resistance underlies many of the challenges that are summarized in Figure 3. The approved targeted therapies for patients with BRAF^V600E^ mutations, as well as NTRK and RET gene fusions, exemplify this. Although many resistance mechanisms are shared, there is growing evidence to suggest that there are cancer-specific factors, although this is not fully understood. Furthermore, the complex interplay between the immune system and tumor cells is key to understanding resistance towards immunotherapy. The diagnostic dilemmas of certain biomarkers, such as TMB-H, are fundamental to discuss due to cancer-specific factors. The addition of the first approved antibody drug conjugate also illustrates the wide clinical benefit of these treatments. Despite the rapidly growing list of approved tissue-agnostic therapies, there are still many gaps in the literature. The expanded indications have provided clinical benefit for patients who otherwise lack alternative options. However, cancer type still plays an imperative role in response rates and survival. Thus, determining whether these treatments are truly histology-agnostic is another challenge to address.

## Figures and Tables

**Table 1:** Summary of tumor-agnostic treatments approved by FDA.

Drug(s)	Approval Date	Trial(s)	Target	Number of Patients	ORR (%) (95% CI)	CR	PR	mDOR (Months)	DOR > 6 Months (%)	DOR > 9 Months (%)	DOR > 12 Months (%)	DOR > 24 Months (%)	Ref.
Pembrolizumab	23 May 2017	KN-012, -016, -028, -158, -164	dMMR MSI-H	149	39.6 (31.7–47.9)	n = 11	n = 48	NE	78	-	-	-	[10]
Larotrectinib	26 November 2018	LOXO-TRK-14001, NAVIGATE, SCOUT	NTRK fusion	55	75 (61–85)	22%	53%	NR	73	63	39	-	[11]
Entrectinib	15 August 2019	ALKA-372-001, STARTRK-1, STARTRK-2	NTRK fusion	54	57 (43–71)	-	-	-	68	-	45	-	[12]
Pembrolizumab	16 June 2020	KN-158	TMB-H (≥10 mut/Mb)	102	29 (21–39)	4%	25%	NR	-	-	57	50	[13]
Dostarlimab	17 August 2021	GARNET	dMMR MSI-H	209	41.6 (34.9–48.6)	9.1%	32.5%	34.7	95.4	-	-	-	[14]
Dabrafenib + trametinib	22 June 2022	NCI-MATCH, BRF117019 (ROAR), CTMT212X2101	BRAF V600E (CRC excluded)	Adults: 131 Children: 36	Adults: 41 (33–50) Children: 25 (12–42)	-	-	-	78	-	-	44	[15]
Selpercatinib	21 September 2022	LIBRETTO-001	RET fusion	41	44 (28–60)	-	-	24.5	67	-	-	-	[16]
Trastuzumab deruxtecan	5 April 2024	DESTINY - PanTumor02 (a), - Lung01 (b), - CRC02 (c)	HER2-positive (IHC3+)	192	51.4 (41.7–61.0) (a) 52.9 (27.8–77.0) (b) 46.9 (34.3–59.8) (c)	-	-	19.4 (a) 6.9 (b) 5.5 (c)	-	-	-	-	[17]
Repotrectinib	13 June 2024	TRIDENT-1	NTRK fusion	88 TKI-pretreated cohort = 48 (a) TKI-naive cohort = 40 (b)	50 (35–65) (a) 58 (41–73) (b)	-	-	9.9 (7.4–13.0) (a) NE (b)	-	-	-	-	[18]

The data for the DESTINY-PanTumor02 trial (a), DESTINY-Lung01 (b), and DESTINY-CRC02 (c) trials are shown with corresponding ORR and mDOR. Abbreviations: 95% confidence interval (95% CI), complete response (CR), colorectal cancer (CRC), deficient mismatch repair (dMMR), duration of response (DOR), human epidermal growth factor receptor 2 (HER2), immunohistochemistry (IHC), Keynote (KN) trials, median duration of response (mDOR), microsatellite instability-high (MSI-H), mutations per megabase (mut/Mb), neurotrophic tyrosine receptor kinase (NTRK), not evaluable (NE), not reached (NR), objective response rate (ORR), partial response (PR), patient population (n), REarranged during Transfection (RET), tumor mutational burden-high (TMB-H), tyrosine kinase inhibitor (TKI). Lessons learned from development of tumor-agnostic therapies.

## Data Availability

No new data were created or analyzed in this study.

## Supplementary Materials

The following supporting information can be downloaded at https://www.mdpi.com/article/10.3390/cancers18101568/s1. Figure S1: Median duration of response observed with (A) Larotrectrectinib in NTRK fusion-positive solid tumors; (B) Entrectinib in NTRK fusion-positive solid tumors; and (C) Selpercatinib in RET fusion-positive solid tumors. Figure S2: Objective response rate observed with Pembrolizumab in dMMR/MSI-H solid tumors. Figure S3: Objective response rates observed with Dostarlimab in MMR deficient solid tumors. Figure S4: Objective response rates observed with Pembrolizumab in TMB-H solid tumors. Figure S5: Median duration of response observed with Trastuzumab deruxtecan in HER2-positive (IHC3+) solid tumors. Table S1: Cancer-specific Data of Tumor-agnostic Treatments.

## Abbreviations

95% CI	95% confidence interval
ADCs	antibody drug conjugates
ALK	anaplastic lymphoma kinase
APC	adenomatous polyposis coli
APM	antigen processing machinery
BRAF	B-Raf proto-oncogene
BRCA1/2	breast cancer gene 1/2
CD8+	cluster of differentiation 8 positive T cells
CRC	colorectal cancer
CR	complete response
CTLA-4	cytotoxic T-lymphocyte–associated protein 4
D + T	dabrafenib plus trametinib
dMMR	deficient mismatch repair
DNA	deoxyribonucleic acid
DOR	duration of response
EC	endometrial cancer
EGFR	epidermal growth factor receptor
ERK	extracellular signal-regulated kinase
FDA	United States Food and Drug Administration
FGFR	fibroblast growth factor receptor
FISH	fluorescence in situ hybridization
HER2	human epidermal growth factor receptor 2
HER3	human epidermal growth factor receptor 3
HRD	homologous recombination deficiency
ICI	immune checkpoint inhibitor
IGF1R	insulin-like growth factor 1 receptor
IHC	immunohistochemistry
IFN-γ	interferon gamma
KRAS	Kirsten rat sarcoma viral oncogene homolog
MAPK	mitogen-activated protein kinase
MDSCs	myeloid-derived suppressor cells
MET	mesenchymal-epithelial transition factor
mCRC	metastatic colorectal cancer
MHC	major histocompatibility complex
MSI	microsatellite instability
MSI-H	microsatellite instability-high
mOS	median overall survival
mPFS	median progression-free survival
NGS	next-generation sequencing
NTRK	neurotrophic tyrosine receptor kinase
NSCLC	non-small cell lung cancer
ORR	objective response rate
PD-1	programmed cell death protein 1
PD-L1	programmed death-ligand 1
PDAC	pancreatic ductal adenocarcinoma
PI3K/AKT	phosphoinositide 3-kinase/protein kinase B pathway
PR	partial response
PTEN	phosphatase and tensin homolog
RAF	rapidly accelerated fibrosarcoma kinase
RAS	rat sarcoma viral oncogene
RET	rearranged during transfection
RTK	receptor tyrosine kinase
SCLC	small cell lung cancer
TAMs	tumor-associated macrophages
TCGA	The Cancer Genome Atlas
T-DXd	trastuzumab deruxtecan
TMB	tumor mutational burden
TMB-H	tumor mutational burden-high
TME	tumor microenvironment
TRK	tropomyosin receptor kinase
Tregs	regulatory T cells
