# Supplementary material for: Understanding the Promise and Challenges of Tumor-Agnostic Therapy: Could One Size Really Fit All?
Source: Cancers (Basel). 2026 May 12;18(10):1568. doi: 10.3390/cancers18101568 (PMC13204736; doi:10.3390/cancers18101568)
Supplement: Supplementary file 1 [file cancers-18-01568-s001.zip › cancers-4283743-supplementary.pdf]

Supplementary Figures

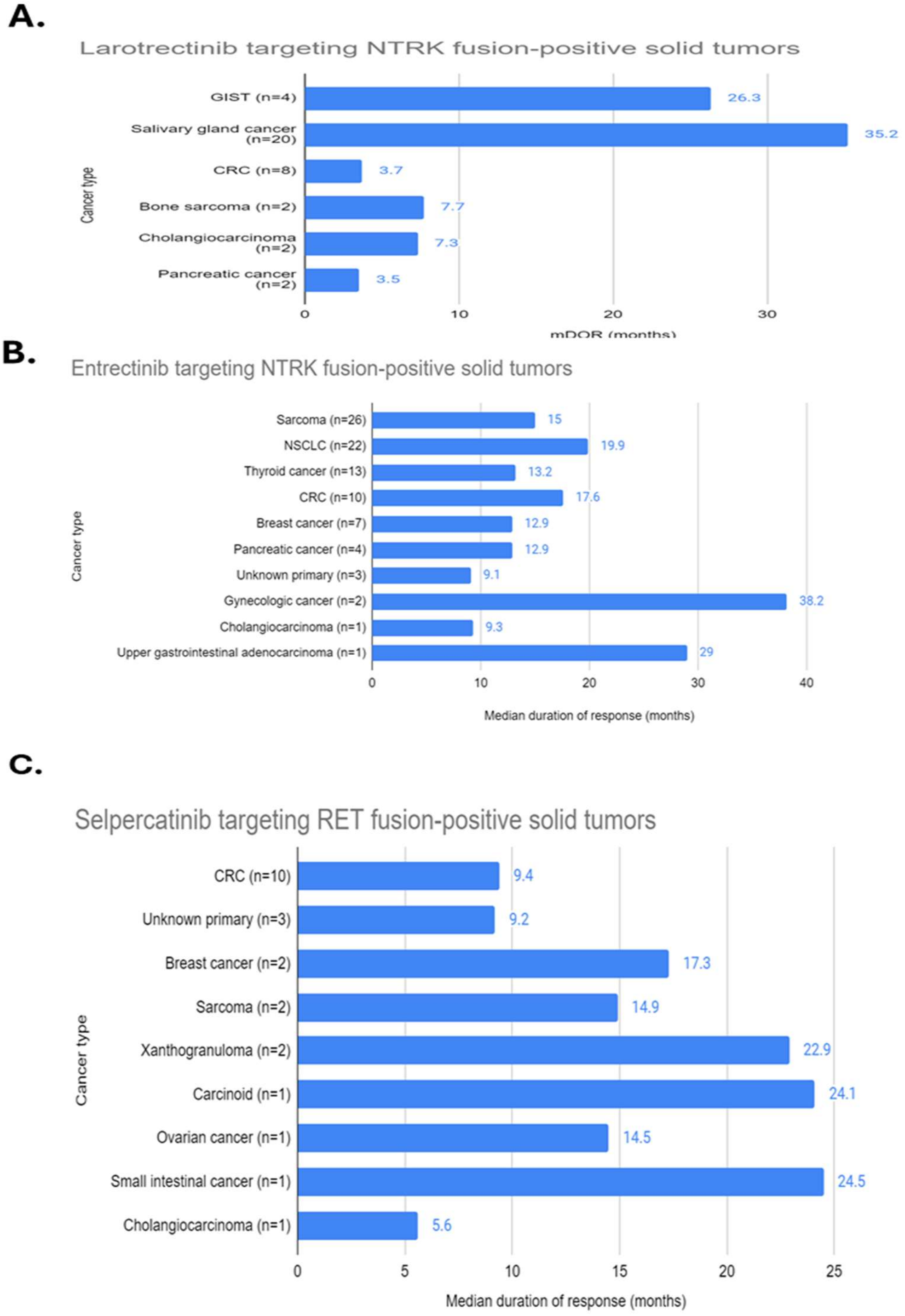

Supplementary Figure S1. Median duration of response observed with A) Larotrectinib in NTRK fusion-positive solid tumors (Reference: [26]**); B) Entrectinib in NTRK fusion-positive solid tumors** (Reference: [28])**; and C) Selpercatinib in RET fusion-positive solid tumors (**Reference: [29]).

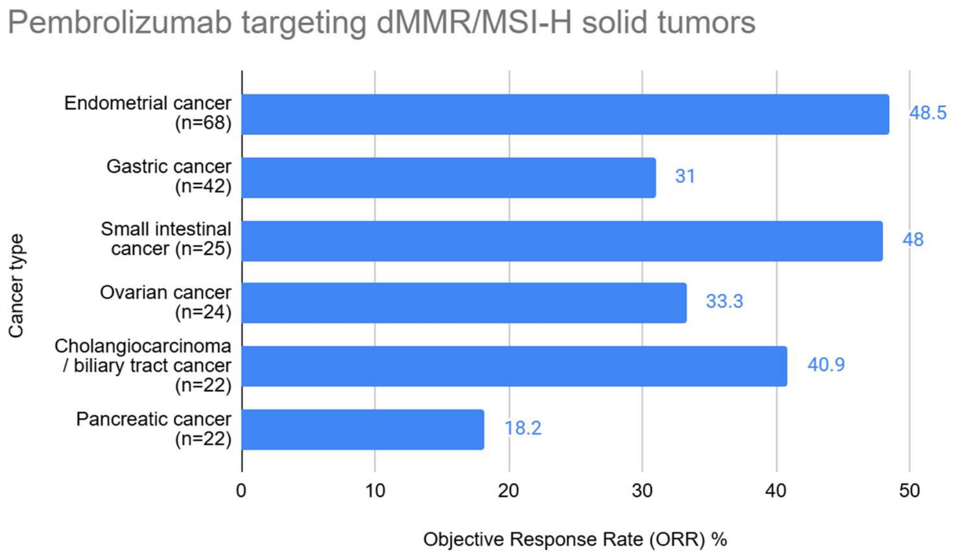

Supplementary Figure S2. Objective response rate observed with Pembrolizumab in dMMR/MSI-H solid tumors (Reference: [32]).

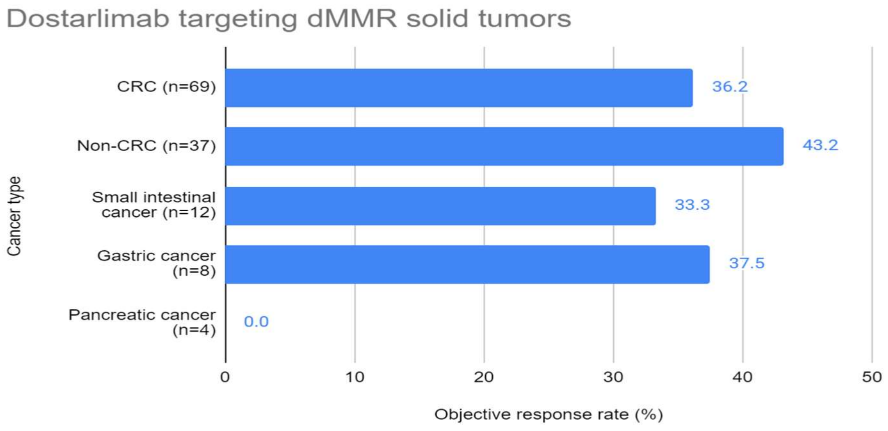

Supplementary Figure S3. Objective response rates observed with Dostarlimab in MMR deficient solid tumors (Reference: [35]).

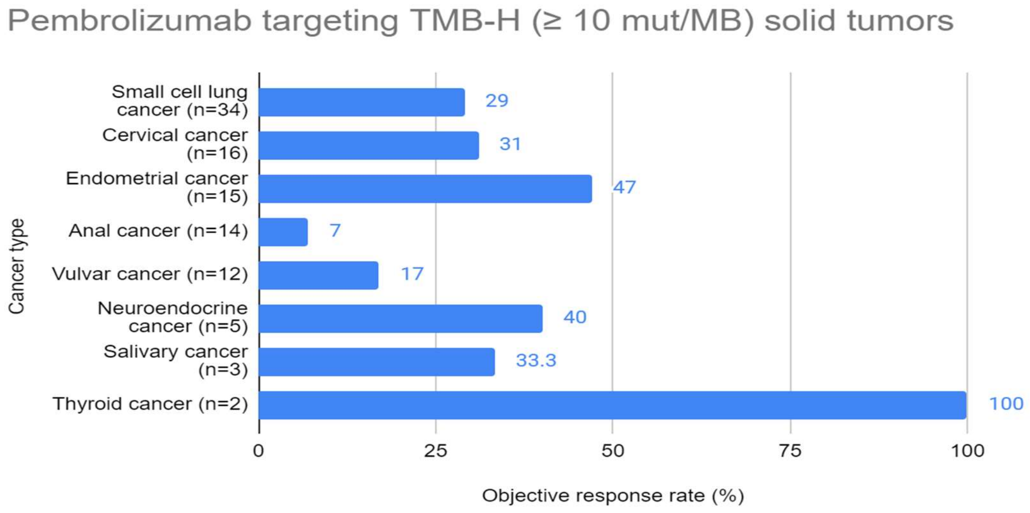

Supplementary Figure S4. Objective response rates observed with Pembrolizumab in TMB-H solid tumors (Reference: [37,38]).

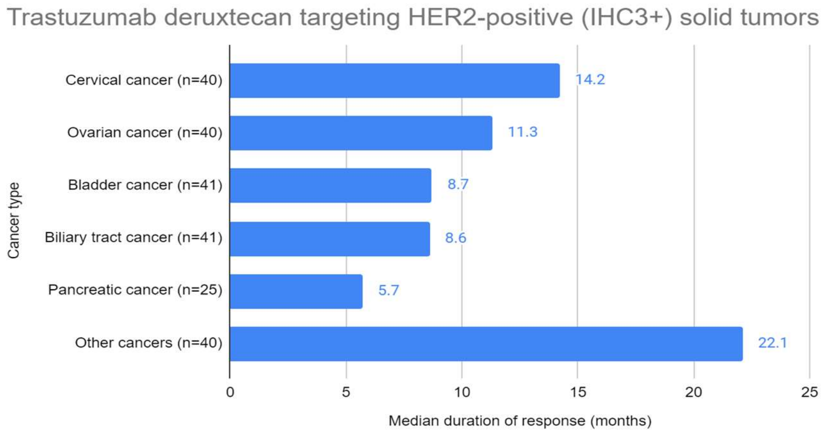

Supplementary Figure S5. Median duration of response observed with Trastuzumab deruxtecan in HER2-positive (IHC3+) solid tumors (Reference: [40]).

Supplementary Table (S1): Cancer-specific Data of Tumor-agnostic Treatments

Drug(s)	Clinical trial(s)	Target & Cancer type	Number of Patients	ORR: % (95% CI)	CR: n (%)	PR: n (%)	mDOR (months) (95% CI)	mPFS (months) (95% CI)	mOS (months) (95% CI)	Reference
Dabrafenib + Trametinib	NCI-MATCH ROAR	**BRAF V600E-positive cancers**	131	41 (33-50)	-	-	NR	-		[21]
		Biliary tract cancer	48	46 (31-61)	-	-	1.8	-		
		High grade glioma	48	33 (20-48)	-	-	3.9	-		
		Low grade glioma	14	50 (23-77)	-	-	6	-		
		Low grade serous ovarian carcinoma	5	80	-	-	12	-		
		Small intestinal adenocarcinoma	4	50	-	-	7	-		
		Pancreatic adenocarcinoma	3	0	-	-	NA	-		
		Adenoneuroendocrine carcinoma	2	0	-	-	NA	-		
		Neuroendocrine carcinoma of the colon	2	0	-	-	NA	-		
		Mandibular ameloblastoma	1	100	-	-	30	-		
		Combined small cell squamous carcinoma of the lung	1	100	-	-	5	-		
		Peritoneal serous adenocarcinoma (mucinous	1	100	-	-	8	-		

Drug(s)	Clinical trial(s)	Target & Cancer type	Number of Patients	ORR: % (95% CI)	CR: n (%)	PR: n (%)	mDOR (months) (95% CI)	mPFS (months) (95% CI)	mOS (months) (95% CI)	Reference
		papillary subtype)								
		Anal cancer	1	0	-	-	NA	-		
		GIST	1	0	-	-	NA	-		
**Dabrafenib + Trametinib**	**ROAR**	**BRAF V600E-positive cancers**	206							[46]
		Anaplastic thyroid cancer	36	53 (35.5-69.6)	2 (6%)	17 (47%)				
		Biliary tract cancer	43	47 (31.2-62.3)	1 (2%)	19 (44%)				
		Small intestinal adenocarcinoma	3	67 (9.4-99.2)	-	2 (67%)				
		Low grade glioma	13	54 (25.1-80.8)	1 (8%)	6 (46%)				
		High grade glioma	45	31 (18.2-46.6)	3 (7%)	11 (24%)				
		Hairy cell leukemia*	55	89 (77.8-95.9)	10 (18%)	13 (24%)				
		Multiple myeloma*	10	50 (18.7-81.3)	0 (0%)	2 (20%)				
		GIST	1	0 (0%)						
**Dabrafenib + Trametinib**	**NCT01072175**	**BRAF V600E-positive metastatic colorectal cancer**	43	12	1 (2%)	4 (9%)		3.5(3.4-4.0)		[45]
**Larotrectinib**	**LOXO-TRK-14001 NAVIGA**	**NTRK fusion-positive**	153	79 (72-85)	24 (16%)	97 (63%)	35.2 (22.8–NE)	28.3 (22·1–NE)	44.4 (36.5–NE)	[26]

Drug(s)	Clinical trial(s)	Target & Cancer type	Number of Patients	ORR: % (95% CI)	CR: n (%)	PR: n (%)	mDOR (months) (95% CI)	mPFS (months) (95% CI)	mOS (months) (95% CI)	Reference
	TE SCOUT	cancers (153)								
		Infantile fibrosarcoma	28	96 (82–100)			NE (NE–NE)			
		GIST	4	100 (40-100)			26.3 (7.6-26.3)			
		Soft tissue sarcomas other than infantile fibrosarcoma or GIST	36	81 (64–92)			NE (10.1–NE)			
		Thyroid cancer	24	79 (58–93)			NE (14.8–NE)			
		Salivary gland cancer	20	90 (68–99)			35.2 (13.3–NE)			
		Lung cancer	12	75 (43–95)			NE (NE–NE)			
		CRC	8	50 (16–84)			3.7 (3.7–NE)			
		Melanoma	7	43 (10–82)			NE (3.7–NE)			
		Breast cancer	4	75 (19–99)			NE (NE–NE)			
		Bone sarcoma	2	50 (1–99)			7.7 (NE–NE)			
		Cholangiocarcinoma	2	50 (1–99)			7.3 (NE–NE)			
		Pancreatic cancer	2	50 (1–99)			3.5 (NE–NE)			
		Appendiceal cancer	1	0 (0%)			–			
		Congenital mesoblastic nephroma	1	100 (3–100)			NE (NE–NE)			
		Hepatocellular cancer	1	0			–			
		Unknown primary	1	100 (3–100)			NE (NE–NE)			

Drug(s)	Clinical trial(s)	Target & Cancer type	Number of Patients	ORR: % (95% CI)	CR: n (%)	PR: n (%)	mDOR (months) (95% CI)	mPFS (months) (95% CI)	mOS (months) (95% CI)	Reference
**Larotrectinib**	**LOXO-TRK-14001 NAVIGATE SCOUT**	**NTRK fusion-positive cancers (180)**<br>Including:<br>• Lung cancer (N=30)<br>• Soft tissue sarcoma (N=30)<br>• Thyroid cancer (N=28)<br>• Salivary gland cancer (N=25)<br>• CRC (N=23)	180	57 (50-65)	29 (16%)	74(41%)	43.3 (29.2-NE)	24.6 (11.3-34.5)	48.7 (38.5-NE)	[27]
**Larotrectinib**	**NCT02637687 NCT02576431**	**NTRK fusion-positive primary CNS cancers**	33	30 (16-49)	3 (9%)	7 (21%)	NR (range: 3.8-22+) (3.8-NE)	18.3 (6.7-NE)	NR (16.9-NE)	[55]
		Pediatric high-grade glioma	13	38 (14-68)						
		Pediatric low-grade glioma	7	43 (10-82)						
**Entrectinib**	**ALKA-372-00 STARTRK-1**	**NTRK fusion-positive cancers**	121	61.2 (51.9-69.9)	19 (15.7%)	55 (45.5%)	20 (13.0-38.2)	13.8 (10.1-19.9)	33.8 (23.4-46.4)	[28]

Drug(s)	Clinical trial(s)	Target & Cancer type	Number of Patients	ORR: % (95% CI)	CR: n (%)	PR: n (%)	mDOR (months) (95% CI)	mPFS (months) (95% CI)	mOS (months) (95% CI)	Reference
	**STARTRK-2**	Sarcoma	26	57.7% (36.9-76.7)			15.0 (4.6-NE)	10.1 (6.3-13.7)	18.7 (14.5-NE)	
		Mammary analog secretory carcinoma (MASC)	24	83.3 (62.6-95.3)			NE (NE-NE)	NE (13.8-NE)	NE (NE-NE)	
		NSCLC	22	63.6 (40.7-82.8)			19.9 (10.4-29.4)	14.9 (6.5-30.4)	NE (20.8-NE)	
		Thyroid cancer	13	53.8 (25.1-80.8)			13.2 (7.9-NE)	19.9 (6.5-33.8)	19.9 (14.5-NE)	
		CRC	10	20(2.5-55.6)			17.6 (15.1-20)	2.8 (1.9-16)	16 (10.8-37.1)	
		Breast cancer	7	71.4 (29.0-96.3)			12.9 (4.2-NE)	10.1 (5.1-NE)	19.2 (5.1-NE)	
		Neuroendocrine tumor	5	40 (5.3-85.3)			NE (11.1-NE)	15.6 (0.9-NE)	40.5 (28.6-40.5)	
		Pancreatic cancer	4	75 (19.4-99.4)			12.9 (7.1-12.9)	12.8 (6.2-17.5)	22.0 (11.2-30.7)	
		Unknown primary	3	33.3 (0.8-90.6)			9.1 (NE-NE)	7.2 (4.4-10.0)	14.3 (NE-NE)	
		Gynecologic cancer	2	50 (1.3-98.7)			38.2 (NE-NE)	27.4 (13.7-41.2)	39.3 (32.1-46.4)	
		Head and neck cancer	2	100 (2.5-100)			NE (16.9-NE)	NE (17.6-NE)	NE (NE-NE)	
		Cholangiocarcinoma	1	100 (2.5-100.0)			9.3 (NE-NE)	12.0 (NE-NE)	23.4 (NE-NE)	
		Upper gastrointestinal adenocarcinoma	1	100 (2.5-100.0)			29.0 (NE-NE)	30.0 (NE-NE)	NE (NE-NE)	

Drug(s)	Clinical trial(s)	Target & Cancer type	Number of Patients	ORR: % (95% CI)	CR: n (%)	PR: n (%)	mDOR (months) (95% CI)	mPFS (months) (95% CI)	mOS (months) (95% CI)	Reference
		Neuroblastoma	1	0				0.1 (NE-NE)	0.1 (NE-NE)	
**Repotrectinib**	**TRIDENT-1**	**NTRK fusion-positive cancers**								[24]
		A. Cohort: TKI-pretreated	69	48 (36-60)	2 (3%)	31 (45%)	9.8 (7.4-13.0)	7.4 (3.9-9.7)	18.6 (11.6-25.3)	
		B. Cohort: TKI-naive	51	59 (44-72)	8 (16%)	22 (43%)	NE	30.3 (9.0-NE)	NR	
		**NSCLC**								
		A. Cohort: TKI-pretreated	17	53 (28-77)						
		B. Cohort: TKI-naive	27	63 (42-81)						
		**Thyroid cancer**								
		A. Cohort: TKI-pretreated	7	29 (4-71)						
		B. Cohort: TKI-naive	6	100 (54-100)						
		**Salivary gland cancer**								
		A. Cohort: TKI-pretreated	12	75 (43-94)						
		B. Cohort: TKI-naive	5	80 (28-100)						
		**Soft tissue sarcoma**								
		A. Cohort: TKI-pretreated	10	10 (0-44)						
		B. Cohort: TKI-naive	3	0 (0-84)						

Drug(s)	Clinical trial(s)	Target & Cancer type	Number of Patients	ORR: % (95% CI)	CR: n (%)	PR: n (%)	mDOR (months) (95% CI)	mPFS (months) (95% CI)	mOS (months) (95% CI)	Reference
		**Breast cancer**								
		A. Cohort: TKI-pretreated	1	100 (2-100)						
		B. Cohort: TKI-naive	2	0 (0-84)						
		**Colorectal cancer**								
		A. Cohort: TKI-pretreated	4	50 (7-93)						
		B. Cohort: TKI-naive	2	50 (1-99)						
		**Cholangiocarcinoma**								
		A. Cohort: TKI-pretreated	2	50 (1-99)						
		B. Cohort: TKI-naive	1	0 (0-98)						
		**Glioblastoma**								
		A. Cohort: TKI-pretreated	3	33 (1-91)						
		B. Cohort: TKI-naive	1	0 (0-98)						
		**Peripheral nerve sheath tumor**								
		A. Cohort: TKI-pretreated	2	100 (16-100)						
		B. Cohort: TKI-naive	1	100 (2-100)						
		**Neuroendocrine tumor**								
		A. Cohort: TKI-	3	100 (29-100)						

Drug(s)	Clinical trial(s)	Target & Cancer type	Number of Patients	ORR: % (95% CI)	CR: n (%)	PR: n (%)	mDOR (months) (95% CI)	mPFS (months) (95% CI)	mOS (months) (95% CI)	Reference
		pretreated								
		B. Cohort: TKI-naive	0	NA						
		**Pancreatic cancer**								
		A. Cohort: TKI-pretreated	3	0 (0-71)						
		B. Cohort: TKI-naive	0	NA						
		**Other cancer types**								
		A. Cohort: TKI-pretreated	5	40 (5-85)						
		B. Cohort: TKI-naive	3	0 (0-71)						
**Selpercatinib**	**LIBRETTO-001**	**RET fusion-positive NSCLC**	316							[25]
		Cohort: treatment-naive	69	84 (73-92)	4 (6%)	54 (78%)	20.1 (13.0-NE)	22.0 (13.8-NE)	NE	
		Cohort: received previous systemic therapy	247	61 (55-67)	18 (7%)	133 (54%)	28.6 (20.4-NE)	24.9 (19.3-NE)	NE	
**Selpercatinib**	**LIBRETTO-001**	**RET fusion-positive thyroid cancer**	19	58 (34–80)	0 (0%)	11 (58%)	NE (9.5–NE)	NE (10.0–NE)		[30]
**Selpercatinib**	**LIBRETTO-001**	**RET fusion-positive solid tumors other than NSCLC and thyroid cancer**	41	43.9 (28.5–60.3)	2 (5%)	16 (39%)	24.5 (9.2–NE)	13.2 (7.4-26.2)	18.0* (10.7-NE)	[29]
		Pancreatic cancer	11	54.5 (23.4-83.3)			NR (NR-NR)			

Drug(s)	Clinical trial(s)	Target & Cancer type	Number of Patients	ORR: % (95% CI)	CR: n (%)	PR: n (%)	mDOR (months) (95% CI)	mPFS (months) (95% CI)	mOS (months) (95% CI)	Reference
		CRC	10	20.0 (2.5-55.6)			9.4 (5.6-13.3)			
		Salivary	4	50.0 (6.8-93.2)			NR (5.7-NR)			
		Unknown primary	3	33.3 (0.8-90.6)			9.2 (NR-NR)			
		Breast cancer	2	100 (15.8-100.0)			17.3 (17.3-17.3)			
		Sarcoma	2	50 (1.3-98.7)			14.9 (NR-NR)			
		Xanthogranuloma*	2	50.0 (1.3-98.7)			22.9 (NR-NR)			
		Carcinoid	1	100 (2.5-100.0)			24.1 (NR-NR)			
		Ovarian cancer	1	100 (2.5-100.0)			14.5 (NR-NR)			
		Small intestinal cancer	1	100 (2.5-100.0)			24.5 (24.5-24.5)			
		Cholangiocarcinoma	1	100.0 (2.5-100.0)			5.6 (NR-NR)			
		Pulmonary carcinosarcoma	1	0 (0.0-97.5)						
		Rectal neuroendocrine	1	0 (0.0-97.5)						
		Carcinoma of the skin	1	0 (0.0-97.5)						
**Selpercatinib**	**LIBRETTO-001**	**RET fusion-positive gastrointestinal cancers (52)**	52	44.2 (30.5-58.7)	3 (5.8%)	20 (38.5%)	37.2 (13.3-NE)	13.2 (5.6-26.2)		[31]
		Pancreatic cancer	13	53.8 (25.1-80.8)	1 (7.7%)	6 (46.2%)	52.1 (2.5-NE)	5.6 (3.2-NE)		

Drug(s)	Clinical trial(s)	Target & Cancer type	Number of Patients	ORR: % (95% CI)	CR: n (%)	PR: n (%)	mDOR (months) (95% CI)	mPFS (months) (95% CI)	mOS (months) (95% CI)	Reference
		CRC	13	30.8 (9.1-61.4)	0 (0%)	4 (30.8%)	13.3 (5.6-NE)	9.1 (4.0-17.0)		
**Pembrolizumab**	**KN-012, -016, -028, -158, -164 (data for accelerated approval)**	**dMMR / MSI-H-positive cancers**	149	39.6 (31.7-47.9)	11 (7%)	48 (32%)	NE (range: 1.6+ - 22.7+)<br>DOR ≥6 months: 78%			[37]
		CRC	90	36						
		Non-CRC	59	46						
**Pembrolizumab**	**KN-051, -158, -164 (data for full approval)**	**dMMR / MSI-H-positive cancers**	504	33.3 (29.2-37.6)	10.3 %	23.0 %	63.2 (range: 1.9+ - 63.9+)<br>DOR ≥12 months: 77%<br>DOR ≥36 months: 39%			[33]
		CRC	124	34 (26-43)			Range: 4.4-58.5			
		Non-CRC	380	33 (28-38)			Range: 1.9-63.9			
**Pembrolizumab**	**KN-158**	**dMMR / MSI-H cancers other than CRC (321)**	321	30.8 (25.8-36.2)	27 (8.4%)	72 (22.4%)	47.5 (range: 2.1+ - 51.1+)	3.5 (2.3-4.2)	20.1 (14.1-27.1)	[32]
		Endometrial cancer	68	48.5 (36.2-61.0)	10 (14.7%)	23 (33.8%)	NR (range: 2.9 - 47.1+)	13.1 (4.9-34.4)	NR (32.4-NR)	
		Gastric cancer	42	31.0 (17.6-47.1)	4 (9.5%)	9 (21.4%)	NR (range: 6.3 - 51.1+)	3.2 (2.1-12.9)	11.0 (5.8-31.5)	
		Small intestinal cancer	25	48.0 (27.8-68.7)	4 (16.0%)	8 (32.0%)	NR (range: 2.1 - 41.8+)	23.4 (4.3-NR)	NR (16.2-NR)	
		Ovarian cancer	24	33.3 (15.6-55.3)	3 (12.5%)	5 (20.8%)	NR (range: 4.2 - 43.5+)	2.2 (2.0-6.2)	33.6 (11.0-NR)	

Drug(s)	Clinical trial(s)	Target & Cancer type	Number of Patients	ORR: % (95% CI)	CR: n (%)	PR: n (%)	mDOR (months) (95% CI)	mPFS (months) (95% CI)	mOS (months) (95% CI)	Reference
		Cholangiocarcinoma / biliary tract cancer	22	40.9 (20.7-63.6)	3 (13.6 %)	6 (27.3 %)	30.6 (range: 6.2 - 40.5+)	4.2 (2.1-24.9)	19.4 (6.5-NR)	
		Pancreatic cancer	22	18.2 (5.2-40.3)	1 (4.5%)	3 (13.6 %)	NR (range: 8.1 - 24.3+)	2.1 (1.9-3.4)	3.7 (2.1-9.8)	
**Dostarlimab**	**GARNET**	**dMMR / MSI-H cancers**	106	38.7 (29.4-48.6)	7.5%		NR			[35]
		CRC	69	36.2 (25.0-48.7)						
		Non-CRC	37	43.2 (27.1-60.5)						
		Small intestinal cancer	12	33.3 (9.9-65.1)						
		Gastric cancer	8	37.5 (8.5-75.5)						
		Pancreatic cancer	4	0 (0.0-60.2)						
		Liver cancer	2	50		50				
		Ovarian cancer	2	50		50				
		Adrenal cortical	1	100		100				
		Biliary neoplasm	1	100	100					
		Breast cancer	1	100	100					
		Esophageal cancer	1	0						
		Gallbladder cancer	1	100	100					
		Genital neoplasm malignant female	1	100		100				
		Pleural	1	100		100				

Drug(s)	Clinical trial(s)	Target & Cancer type	Number of Patients	ORR: % (95% CI)	CR: n (%)	PR: n (%)	mDOR (months) (95% CI)	mPFS (months) (95% CI)	mOS (months) (95% CI)	Reference
		Renal cell carcinoma	1	0						
		Unknown origin	1	100		100				
**Dostarlimab**	**GARNET**	**dMMR-positive cancers (327)**	327	44.0 (38.6-49.6)	43 (13.1 %)	101 (30.9 %)	NR (range: ≥1.18 to ≥47.21)	6.9 (4.2-13.6)	NR (31.6-NR)	[69]
**Dostarlimab**	**GARNET**	**dMMR/MSI-H or POLE-mutated cancers**	347	44.1(38.8-49.5)	46 (13.3 %)	107 (30.8 %)	NR (NR-NR)	7.0 (4.2-13.8)	NR (39.9-NR)	[69]
		Endometrial cancer	143	45.5 (37.1-54.0)	23 (16.1 %)	42 (29.4 %)	NR (38.9-NR)	6.0 (4.1-18.0)	NR (25.7-NR)	
		Non-endometrial cancer	204	43.1 (36.2-50.2)	23 (11.3 %)	65 (31.9 %)	NR (NR-NR)	7.1 (3.6-19.5)	NR (31.5-NR)	
		CRC	115	43.5 (34.3-53.0)	14 (12.2 %)	36 (31.3 %)	NR (NR-NR)	8.4 (3.4-NR)	NR (NR-NR)	
		Gastric cancer	22	45.5 (24.4-67.8)	1 (4.5%)	9 (40.9 %)	NR (17.5-NR)	5.5 (2.8-NR)	20.1 (6.7-NR)	
		Small intestinal cancer	23	39.1 (19.7-61.5)	5 (21.7 %)	4 (17.4 %)	NR (8.3-NR)	8.1 (2.5-16.5)	31.6 (8.2-NR)	
		Pancreatic carcinoma	12	41.7 (15.2-72.3)	0 (0%)	5 (41.7 %)	NR (NR-NR)	3.3 (2.6-NR)	12.7 (3.1-NR)	
		Ovarian cancer	7	42.9 (9.9-81.6)	0 (0%)	3 (42.9 %)				
		Other	25	44.0 (24.4-65.1)	3 (12.0 %)	8 (32.0 %)	4.5 (2.5-NR)	NR (8.8-NR)	NR (13.5-NR)	
**Dostarlimab**	**NCT04165772**	**dMMR/MSI-H rectal adenocarcinoma (12)**	12	100 (74-100)	12 (100 %)					[36]

Drug(s)	Clinical trial(s)	Target & Cancer type	Number of Patients	ORR: % (95% CI)	CR: n (%)	PR: n (%)	mDOR (months) (95% CI)	mPFS (months) (95% CI)	mOS (months) (95% CI)	Reference
**Pembrolizumab**	**KN-158**	**tTMB-H (≥ 10 mut/MB) cancers**	102	29.4 (20.8-39.3)	4 (3.9%)	26 (25.5%)	NR (22-24.8)	2.1	11.7 (9.1-19.1)	[37,38]
		Small cell lung cancer	34	29 (15-47)			Range: 4.1-32.5+		9.4 (5.6-19.1)	
		Cervical cancer	16	31 (11-59)			Range: 3.7+ - 34.8+		16.7 (7.4-NA)	
		Endometrial cancer	15	47 (21-73)			Range: 8.4+ - 33.9+		22.7 (2.5-NA)	
		Anal cancer	14	7 (0.2-34)			Range: 18.8+		9.0 (3.7-NA)	
		Vulvar cancer	12	17 (2-48)			Range: 8.8-11.0		10.8 (2.2-NA)	
		Neuroendocrine cancer	5	40 (5-85)			Range: 2.2+ - 32.6+		37.2 (7.5-37.2)	
		Salivary cancer	3	33.3		33.3	Range: 31.3+		NR (15.9-NA)	
		Thyroid cancer	2	100	n=2		Range: 8.2-33.2+		NR (29.2-NA)	
		Mesothelioma	1	0					2.9 (NA-NA)	
**Pembrolizumab**	**KN-158**	**Non-tTMB-H (< 10 mut/MB) cancers**	688	6.3 (4.6-8.3)	11 (2%)	32 (5%)	33.1 (range: 4-35.7)	2.1	12.8 (11.1-14.1)	
		Small cell lung cancer	42						6.3 (3.9-7.7)	
		Cervical cancer	59						9.4 (7.7-13.1)	
		Endometrial cancer	67						10.3 (7.9-14.9)	

Drug(s)	Clinical trial(s)	Target & Cancer type	Number of Patients	ORR: % (95% CI)	CR: n (%)	PR: n (%)	mDOR (months) (95% CI)	mPFS (months) (95% CI)	mOS (months) (95% CI)	Reference
		Anal cancer	75						12.0 (8.6-15.7)	
		Vulvar cancer	59						5.3 (3.6-9.5)	
		Neuroendocrine cancer	82						26.4 (19.1-34.4)	
		Salivary cancer	79						18.7 (11.1-24.8)	
		Thyroid cancer	78						34.1 (17.6-NA)	
		Mesothelioma	84						10.4 (7.8-13.6)	
		Biliary cancer	63						9.4 (5.6-11.0)	
**Pembrolizumab**	**KN-158**	**TMB-H (≥ 10 mut/MB and <13 mt/Mb) cancers**	32	13 (4-29)	n=2	n=2				
**Pembrolizumab**	**KN-158**	**TMB-H (≥ 13 mut/MB) cancers**	70	37 (26-50)						
**Trastuzumab Deruxtecan**	**DESTINY-PanTumor02**	**HER2-expressing (IHC3+/2+) cancers**	267	37.1 (31.3-43.2)			11.8 (95% CI 9.6-17.8)	6.9 (95% CI 5.6-8)	13.4 (95% CI 11.9-15.5)	[40]
		Endometrial cancer	40	57.5 (40.9-73.0)	8 (20.0%)	15 (37.5%)	NR (9.9-NR)	11.1 (7.1-NR)	26.0 (12.8-NR)	
		Cervical cancer	40	50.0 (33.8-66.2)	2 (5.0%)	18 (45.0%)	14.2 (4.1-NR)	7.0 (4.2-11.1)	13.6 (11.1-NR)	
		Ovarian cancer	40	45.0 (29.3-61.5)	4 (10.0%)	14 (35.0%)	11.3 (4.1-22.1)	5.9 (4.0-8.3)	13.2 (8.0-17.7)	

Drug(s)	Clinical trial(s)	Target & Cancer type	Number of Patients	ORR: % (95% CI)	CR: n (%)	PR: n (%)	mDOR (months) (95% CI)	mPFS (months) (95% CI)	mOS (months) (95% CI)	Reference
		Bladder cancer	41	39.0 (24.2-55.5)	1 (2.4%)	15 (36.6%)	8.7 (4.3-11.8)	7.0 (4.2-9.7)	12.8 (11.2-15.1)	
		Biliary tract cancer	41	22.0 (10.6-37.6)	1 (2.4%)	8 (19.5%)	8.6 (2.1-NR)	4.6 (3.1-6.0)	7.0 (4.6-10.2)	
		Pancreatic cancer	25	4.0 (0.1-20.4)	0 (0%)	1 (4.0%)	5.7 (NR-NR)	3.2 (1.8-7.2)	5.0 (3.8-14.2)	
		Other cancers	40	30.0 (16.6-46.5)	0 (0%)	12 (30.0%)	22.1 (4.1-NR)	8.8 (5.5-12.5)	21.0 (12.9-24.3)	
**Trastuzumab Deruxtecan**	**DESTINY-PanTumor02**	**HER2-positive (IHC3+) cancers**	75							
		Endometrial cancer	13	84.6 (54.6-98.1)						
		Cervical cancer	8	75.0 (34.9-96.8)						
		Ovarian cancer	11	63.6 (30.8-89.1)						
		Bladder cancer	16	56.3 (29.9-80.2)						
		Biliary tract cancer	16	56.3 (29.9-80.2)						
		Pancreatic cancer	2	0						
		Other cancers	9	44.4 (13.7-78.8)						
**Trastuzumab Deruxtecan**	**DESTINY - Lung01**	**HER2 - overexpressed metastatic NSCLC**	90							[41]
		Cohort receiving 5.4 mg/kg	41	34.1 (20.1-50.6)	2 (5%)	12 (29%)	6.2 (4.2-9.8)	6.7 (4.2-8.4)	11.2 (8.4-NE)	
		Cohort	49	26.5	0	13	5.8 (4.3-NE)	5.7 (2.8-	12.4	

Drug(s)	Clinical trial(s)	Target & Cancer type	Number of Patients	ORR: % (95% CI)	CR: n (%)	PR: n (%)	mDOR (months) (95% CI)	mPFS (months) (95% CI)	mOS (months) (95% CI)	Reference
		receiving 6.4 mg/kg		(15.0–41.1)	(0%)	(27%)		7.2)	(7.8-17.2)	
**Trastuzumab Deruxtecan**	**DESTINY - CRC02**	**HER2-positive metastatic CRC (122)**	122							[42]
		Cohort receiving 5.4 mg/kg	82	37.8 (27.3-49.2)		82 (100 %)	5.5 (4.3-8.1)	5.8 (4.6-7.0)		
		Cohort receiving 6.4 mg/kg	40	27.5 (14.6-43.9)		40 (100 %)	5.5 (3.7-NE)	5.5 (4.2-7.0)		
**Trastuzumab Deruxtecan**	**DESTINY - Gastric02**	**HER2-positive gastric / gastroesophageal junction cancer**	79	42 (30.8–53.4)	4 (5%)	29 (37%)	8.1 (5.9–NE)	5.6 (4.2–8.3)	12.1 (9.4–15.4)	[43]

Abbreviations: 95% confidence interval (95% CI), colorectal cancer (CRC), complete response (CR), deficient mismatch repair (dMMR), duration of response (DOR), gastrointestinal stromal tumor (GIST), human epidermal growth factor receptor 2 (HER2), immunohistochemistry (IHC), Keynote (KN) trials, median duration of response (mDOR), median overall survival (mOS), median progression-free survival (mPFS), microsatellite instability high (MSI-H), mutations per megabase (mut/Mb), neurotrophic tyrosine receptor kinase (NTRK), non-small-cell lung cancer (NSCLC), not available (NA), not evaluable (NE), not reached (NR), objective response rate (ORR), partial response (PR), patient population (n), polymerase epsilon (POLE), REarranged during Transfection (RET), tissue TMB-H (tTMB-H), tumor mutational burden high (TMB-H), tyrosine kinase inhibitor (TKI)

**based on investigator assessment; independent assessment data unavailable*
+ last assessment revealed no progressive disease
